# Psychogenic nonepileptic seizures: clinical characteristics and outcome

**DOI:** 10.1002/brb3.2567

**Published:** 2022-04-12

**Authors:** Bastian Volbers, Katrin Walther, Katrin Kurzbuch, Laura Erdmann, Stephanie Gollwitzer, Johannes D. Lang, Müjgan Dogan Onugoren, Michael Schwarz, Stefan Schwab, Hajo M. Hamer

**Affiliations:** ^1^ Department of Neurology University of Erlangen–Nuremberg Erlangen Germany; ^2^ Epilepsy Center Department of Neurology University of Erlangen–Nuremberg Erlangen Germany

**Keywords:** activities of daily living, mental disorders, nonepileptic seizures, patient‐relevant outcome, prognosis

## Abstract

**Background:**

Clinical characteristics, outpatient situation, and outcome in patients with psychogenic nonepileptic seizures (PNES) remain to be elucidated.

**Methods:**

Patients diagnosed with PNES after video‐electroencephalography (EEG) monitoring (VEM) 03/2000–01/2016 at the Erlangen Epilepsy Center were surveyed between June 2016 and February 2017. Primary outcome was PNES cessation defined as no PNES episodes within > = 12 months prior to the interview. Secondary outcome variables included quality of life (QoL) and dependency. Sensitivity analysis included patients with proven PNES during VEM without comorbid epilepsy.

**Results:**

Ninety‐nine patients were included (median age 38 (interquartile range (IQR 29–52)) years; 68 (69%) females, follow‐up 4 (IQR 2.1–7.7) years). Twenty‐eight (28%) patients suffered from comorbid epilepsy. Twenty‐five (25%) patients reported PNES cessation. Older age at symptom onset (odds ratio (OR) related to PNES cessation: 0.95 (95% CI 0.90–0.99)), comorbid epilepsy (OR 0.16 (95% CI 0.03–0.83)), anxiety disorder (OR 0.15 (95% CI 0.04–0.61)), and tongue biting (OR 0.22 (95% CI 0.03–0.91)) remained independently associated with ongoing PNES activity after adjustment. Sensitivity analysis (*n* = 63) revealed depressive disorder (OR 0.03 (95% CI 0.003–0.34)) instead of anxiety as independent predictor, while this seemed relevant only in patients older than 26 years at onset (OR 0.04 (95% CI 0.002–0.78) versus OR 0.21 (95% CI 0.02–1.84) in patients  younger than 26 years). PNES cessation was associated with increased median QoL (8 (IQR 7–9) versus 5.5 (IQR 4–7); *p* < .001) and an increased frequency of financial independency (14 (56%) versus 21 (28%); *p* = .01).

**Conclusions:**

We found poor outcomes in PNES especially in older patients at onset with comorbid depressive disorder. Comorbid epilepsy also seems to be a major risk factor of ongoing PNES activity, which in turn affects patients’ daily living.

## Graphical abstract

1. Psychogenic nonepileptic seizures (PNES) represent a heterogeneous entity associated with poor long‐term outcome in terms of PNES cessation.
2. PNES cessation is associated with improved quality of life and financial independency.
3. Older age at onset, depressive disorders (especially in late‐onset PNES), anxiety, and comorbid epilepsy are associated with PNES activity and seem to represent distinct etiological mechanisms.
4. Outpatient care is poor in general.
5. “Natural” course of disease activity after diagnosis in patients with PNES cessation (figure):

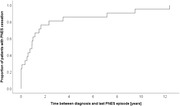

## 1 INTRODUCTION

Psychogenic nonepileptic seizures (PNES) appear as paroxysmal and time‐limited episodes of involuntarily altered or disturbed movement, sensation, or behavior. Cognitive, emotional, and autonomic functions including consciousness may be involved. While the estimated prevalence is 5–20/100,000 (Hingray et al., 2018), about 12%–20% of patients in epilepsy clinics are diagnosed with PNES (Angus‐Leppan, 2008). In contrast to epileptic seizures, PNES are not related to abnormal electric discharges in the brain but to pathopsychological processes (Bodde et al., 2009; Lesser, 1996; Reuber et al., 2003). Thus, PNES represent a distinct entity, even if epileptic seizures may coexist (Benbadis & Sutton, 2015; Bodde et al., 2009; Brigo & Lattanzi, 2019). However, symptom activity does not respond to or may even be worsened by antiseizure medication (ASM) (Niedermeyer et al., 1970; Oto et al., 2005). Existing research indicates a poor outcome regarding disease activity and dependency (Reuber et al., 2003). Only limited knowledge about prognostic factors exists (Bodde et al., 2009) since PNES represent not just one discrete disorder but may be associated with heterogeneous psychological disorders (Kanner et al., 1999), social conflicts, or epilepsy (Reuber & House, 2002). Especially age at symptom onset (Irwin et al., 2000; Reuber et al., 2003), prominent clinical features (Selwa et al., 2000), and ongoing depressive and personality disorders may be related to ongoing PNES activity (Kanner et al., 1999) with pronounced outcome differences depending on the underlying psychopathology (Bodde et al., 2009; Kanner et al., 1999). Research regarding the outcome predictive value of additional epilepsy revealed conflicting results (Jones et al., 2010; Kanner et al., 1999; Reuber et al., 2003; Sadan et al., 2016; Walczak et al., 1995). Finally, the value of PNES cessation as reliable outcome variable has been questioned (Reuber et al., 2005). The aim of the present study was to assess long‐term follow‐up outcomes in PNES patients, their relevance in daily life, and prognostic factors.

## 2 METHODS

This study was approved by our institutional review board (IRB; Friedrich‐Alexander University of Erlangen‐Nuremberg). Verbal informed consent was obtained from each patient in accordance with the IRB approval.

### 2.1 Patients

We included all patients with confirmed PNES diagnosis during Video‐EEG‐monitoring (VEM) after having recorded a typical event without ictal EEG chances (epileptiform activity) between March 2000 and January 2016. In this case, comorbid epilepsy was no exclusion criterion. We also included patients if diagnosis was based on a description of typical episodes including typical PNES characteristics (e.g., long duration, alternating symptoms, “generalized” symptoms in responsive patients) either by the patient or by next of kin without any hints for an existing epilepsy during a detailed work‐up including at least 7–10 days of VEM, cerebral magnetic resonance imaging, and neuropsychological examination (Bodde et al., 2009). Diagnosis of comorbid epilepsy was based on reported or observed symptoms consistent with epileptic seizures in combination with (inter‐)ictal epileptiform activity on EEG recordings. A sensitivity analysis included only patients with proven PNES during VEM without comorbid epilepsy (PNES only). The treating physician discussed the diagnosis with the patient before discharge. A presentation at a psychiatrist or clinical psychologist was recommended. Furthermore, it was recommended to withdraw ASM if VEM uncovered PNES only.

### 2.2 Data assessment

Between June 2016 and February 2017, patients were contacted via mail and informed about the study. A few weeks later, we contacted each patient via telephone to ask for consent to participate. Afterward, a questionnaire was completed on the phone. If a patient preferred to complete the questionnaire without assistance, the questionnaire was sent to the patient via mail. The questionnaire included questions regarding comorbidities including psychiatric diagnoses, comorbid epilepsy, evolution of PNES including the landmarks “occurrence of first episodes” and “time of diagnosis,” description of episodes (positive motor (rigidity, shaking), negative motor (weakness, collapse), purely sensory features or loss of consciousness) and accompanying symptoms including loss of urine/feces or tongue bite, quality of life (QoL; assessed using the 10‐point Likert scale (QOLIE 31) (Cramer et al., 1998) ranging from 1 to 10), dependence on third parties regarding activity of daily living (ADL), governmental support, performed therapies, and patient's self‐rating of all aspects mentioned so far and their relation to PNES. Psychological and psychosocial aspects are reported elsewhere (Walther et al., 2019; Walther et al., 2020). A “dissociative status” was defined as an episode lasting 30 minutes or longer (Reuber et al., 2003). Reported information regarding psychiatric diagnosis, comorbid epilepsy, and PNES features was verified by patients’ records.

### 2.3 Outcome

Primary outcome variable was cessation of PNES defined as a period of at least 1 year without any PNES activity prior to the interview (dichotomized outcome variable). Secondary outcome variables included QoL (ordinal), dependence on financial support (e.g., welfare aid, unemployment benefit, payments of social security), and ADL support and current situation (medication, regular consults) at the time of the interview.

### 2.4 Statistics

We performed statistical analyses using IBM® SPSS® Statistics 21 software package (IBM Corporation, Armonk, NY). Data are presented as mean and standard deviation (SD), median and interquartile range (IQR), or *n* (%) as appropriate. We examined normally distributed interval data (according to Kolmogorov‐Smirnov Test) using independent *t* test and other interval and ordinal data using Wilcoxon Rank Sum test. We used χ^2^ test to analyze nominal data. Univariate *p*‐values were corrected according to Bonferroni‐Holms to account for multiple testing if applicable. We included all meaningful variables with a univariate trend (*p* < .2) toward an association with PNES cessation in a multivariable logistic regression model (both backward and forward LR) (Hosmer et al., 2013; Lemeshow & Hosmer, 2008). We applied the same method (forced entry) to adjust the association of PNES cessation with dichotomized QoL according to the median split method. A Cox‐regression was performed to examine time between diagnosis and last PNES episode in patients with PNES cessation. We used a receiver operating characteristic (ROC) analysis to assess the optimal age cut‐off regarding prediction of PNES cessation. In case of missing data, we indicated the number of included patients for respective analyses. The significance level was set at α = 0.05. Statistical tests were two‐sided.

## 3 RESULTS

### 3.1 Study population

We identified 196 patients. We could not obtain any response from 46 patients (23%) due to incorrect contact information. Forty‐seven patients (24%) declined to participate and four patients (2%) had died (one suicide; three patients were found dead (an association with epileptic seizures was suspected (*n* = 1); due to unknown reasons (*n* = 2)); no further data could be obtained (*n* = 1)) leading to 99 included patients. Median time between diagnosis of PNES and study initiation was longer in patients who did not participate (6.7 years (IQR 3.2–10.1 vs. 4.0 years (IQR 2.1–7.5), *p* = .002). Age at the time of study initiation (42 years (IQR 28–53) and 39 years (IQR 29–52), *p* = .65), female sex (74 (80%) and 68 (69%), *p* = .15), age at the time of PNES diagnosis (35 years (IQR 21–45) and 33 years (IQR 23–47), *p* = .42), and the percentage of patients with additional epilepsy (21 (23%) and 28 (28%), *p* = .32) did not differ between nonparticipating and participating patients.

### 3.2 Patients’ characteristics

For detailed characteristics, please see Table 1. *P*‐values given in the tables are not corrected. In case of a correction according to Bonferroni‐Holms, corrected *p*‐values are given in the text later.

**TABLE 1:** Characteristics of included cohorts

		Total cohort (*N* = 99)			Sensitivity cohort – PNES only (*N* = 63)		
	All patients (*n* = 99)	Ongoing PNES activity (*n* = 74)	No PNES activity ≥1 year (“PNES cessation”; *n* = 25)	*p*‐value (not corrected)	Ongoing PNES activity (*n* = 42)	No PNES activity ≥1 year (“PNES cessation”; *n* = 21)	*p*‐value (not corrected)
Age at interview [years] (IQR)	38 (29‐52)	43 (31–53)	32 (25–41)	.01*	45 (34–53)	32 (27–41)	.01*
Age at first PNES episode [years] (IQR)	25 (18‐38)	29 (18–39)	19 (17–30)	.04*	34 (18–41)	18 (17–30)	.01*
Age at diagnosis [years] (IQR)	32 (22‐46)	36 (26–49)	23 (19–36)	.002*	40 (28–49)	26 (19–36)	.002*
Female sex (%)	68 (69)	51 (69)	17 (68)	.99	31 (74)	14 (67)	.56
Time between diagnosis and interview (Follow‐Up) [years] (IQR)	4 (2.1–7.7)	4.1 (2.1–7.6)	3.6 (2.1–9.6)	.62	3.58 (1.97–7.43)	3.59 (1.87–11.42)	.36
Time between first PNES episode and diagnosis [years] (IQR)	3.7 (1–9.9)	5.2 (1.5–10.8)	1.6 (0.7–4.0)	.03*	6.4 (1.8–10.9)	1.7 (0.7–7.0)	.04*
Time between first PNES episode and interview [years] (IQR)	10 (5.4–17.7)	11 (5.8–17.5)	8.4 (3.9–18.8)	.31	11.1 (7.4–16.5)	10.2 (3.9–19.3)	.86
Comorbid epilepsy (%)	28 (28)	26 (35)	2 (8)	.01*	–	–	–
Depressive/bipolar disorder (%)	64 (65)	50 (68)	14 (56)	.3	36 (86)	10 (48)	.002*
Anxiety disorder (%)	37 (37)	32 (43)	5 (20)	.06	20 (48)	5 (24)	.1
Posttraumatic stress disorder (%)	36 (36)	29 (39)	7 (28)	.35	21 (50)	5 (24)	.06
Suicide attempt or suicidal ideation in history (%)	62 (63; *n* = 87)	48 (73; *n* = 87)	14 (67; *n* = 87)	.59	29 (76; *n* = 56)	11 (61; *n* = 56)	.34
Loss of consciousness during PNES episode (%)	62 (63; *n* = 97)	45 (63; *n* = 97)	17 (68; *n* = 97)	.64	29 (71; *n* = 62)	13 (62; *n* = 62)	.57
Motor symptoms during PNES episode (%)	85 (86)	66 (89)	19 (76)	.18	36 (86)	15 (71)	.19
Loss of urine/feces during PNES episode (%)	29 (29; *n* = 91)	24 (36; *n* = 91)	5 (21; *n* = 91)	.21	14 (36; *n* = 59)	4 (20; *n* = 59)	.25
Tongue biting during PNES episode (%)	31 (31)	28 (38)	3 (12)	.02*	17 (41)	2 (10)	.02*
“Dissociative status” in history (%)	41 (41)	34 (46)	7 (28)	.16	22 (52)	7 (33)	.19

*Note*. Total cohort (*n* = 99, left column); comparison of patients with ongoing psychogenic nonepileptic seizures (PNES) activity and PNES cessation in total cohort (*n* = 99; columns 2–4) and sensitivity cohort (*n* = 63; columns 5–7)). Sensitivity cohort includes only patients with proven PNES during Video‐EEG‐monitoring without additional epileptic seizures. A “dissociative status” was defined as an episode lasting 30 minutes or longer. If patients did not provide any information, included number is indicated for respective variables. Data are given as number and percentage (χ^2^ test or Fisher's Exact Test) or median and interquartile range (IQR; Wilcoxon Rank Sum test). *P*‐values < 0.05 are labeled with asterisk (*).

Abbreviations: IQR,  interquartile range; PNES, psychogenic nonepileptic seizures.

#### 3.2.1 Total cohort (*n* = 99)

Twenty‐five patients (25%) reported PNES cessation according to study definition. Patients with ongoing PNES activity reported a median of two episodes per month (IQR 1–8.3), were older at the time of PNES onset and time of diagnosis, and had more often comorbid epileptic seizures than patients with PNES cessation. Time between first PNES episode and diagnosis was shorter in patients with PNES cessation. After correction according to Bonferroni‐Holms, psychiatric comorbidities (anxiety (corrected *p* = .17) and a tongue biting (corrected *p* = .12) did not differ between both groups.

#### 3.2.2 Sensitivity cohort (*n* = 63)

**FIGURE 1:**
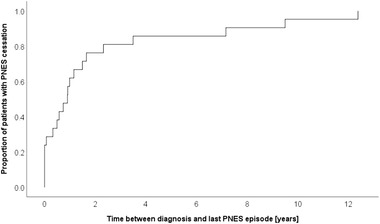
Time between diagnosis and last PNES episode in patients with PNES cessation (*n* = 21). Time between diagnosis and last PNES episode in patients with PNES cessation defined as no PNES activity during > = 12 months prior to the interview (sensitivity analysis, *n* = 21). About 50% of patients reported their last PNES episode within 1 year after diagnosis and about 80% within 2 years after diagnosis

For the sensitivity analysis, 28 patients with comorbid epileptic seizures and 8 patients without proven PNES during VEM were excluded. Patients with ongoing PNES activity were older at the time of first PNES episodes and time of diagnosis and showed tongue biting more often. In addition, time between first PNES episode and diagnosis was longer in those patients than in patients with PNES cessation. In patients with ongoing PNES activities there was a trend toward reporting of depressive disorders (corrected *p* = .09). The way of communicating the diagnosis with patients (including the following items: doctor took time to communicate diagnosis, communication of diagnosis during round, “on the fly” or in elaborate appointment, patient could see video of PNES episode, patient felt being taken seriously) did not correlate with PNES cessation. Whether a patient considered the diagnosis correct or positive was not associated with PNES cessation (data not shown). Figure 1 shows the natural course when patients had their last PNES episode after diagnosis in patients with PNES cessation.

### 3.3 Patients with PNES plus epilepsy versus PNES only

Detailed data are given in Table 2. Patients with comorbid epileptic seizures (*n* = 28) did not differ from patients without additional epileptic seizures (*n* = 63) regarding median age and female sex. They reported less often a depressive/bipolar disorder (corrected *p* = .04). Only one patient (4%) considered epileptic seizures a major health problem. Patients with additional epilepsy rated the diagnosis “PNES” less often positive than patients without additional epilepsy. Furthermore, they stated to show motor symptoms as main symptom during an attack they considered a PNES episode more often (corrected *p* = .04), while frequency of loss of consciousness, loss of urine/feces, and tongue bite during episodes did not differ between both groups. Median QoL did also not differ between both groups, while patients with comorbid epilepsy showed less often a PNES cessation. Patients with comorbid epilepsy less often had a driver's license, were less often allowed to conduct a vehicle at the time of the interview, and were classified with a higher median percentage of disability. Independency of governmental financial support did not differ between both groups. Patients with comorbid epilepsy more often received ASM (corrected *p* < .001), while frequency of antidepressants and benzodiazepine intake did not differ (corrected *p* = .576 and *p* = .29, respectively). One‐third of patients with comorbid epilepsy reported regular consults at an epilepsy center (nine (33%) vs. four (7%) patients without comorbid epilepsy, corrected *p* = .01). All other characteristics did not differ between patients with and without comorbid epilepsy (such as anxiety disorder or time between onset, diagnosis, and follow‐up; data not shown).

**TABLE 2:** Characteristics of patients with proven psychogenic nonepileptic seizures only (PNES, sensitivity cohort, *n* = 63) compared to patients with PNES and additional epileptic seizures (*n* = 28)

	PNES patients without comorbid epilepsy (sensitivity cohort, *n* = 63)	PNES patients with comorbid epilepsy (*n* = 28)	*p*‐value (not corrected)
Age at interview [years] (IQR)	41 (31–53)	37 (25–53)	.78
Age at first PNES episode [years] (IQR)	30 (18–39)	23 (17–36)	.57
Age at diagnosis [years] (IQR)	34 (26–46)	30 (21–46)	.51
Female sex (%)	45 (71)	15 (54)	.1
Depressive/bipolar disorder (%)	46 (73)	13 (46)	.01*
Loss of consciousness during PNES episode (%, *n* = 89)	42 (68)	14 (52)	.15
Motor symptoms during PNES episode (%, *n* = 90)	42 (68)	25 (89)	.04*
Loss of urine/feces during PNES episode (%, *n* = 84)	18 (31)	9 (36)	.8
Tongue biting during PNES episode (%)	19 (30)	12 (43)	.34
“Dissociative status” in history (%)	29 (46)	9 (32)	.26
PNES is considered as major medical issue (%)	34 (54)	19 (68)	.26
PNES diagnosis is rated positive (%, *n* = 76)	21 (41)	3 (12)	.02*
Quality of life (IQR)	7 (4–8)	6.8 (5–7.9)	.97
No PNES activity ≥1 year (“PNES cessation”, %)	21 (33)	2 (7)	.008*
Percentage of severe disability [%] (IQR, *n* = 84)	50 (38–80)	75 (50–100)	.02*
Driver's license (%)	46 (73)	14 (50)	.03*
Patients allowed conducting a vehicle at time of interview (%)	23 (37)	2 (7)	.004*
No governmental financial support necessary (%)	24 (38)	7 (25)	.24
Anti‐seizure medication (%)	19 (30)	27 (96)	<.001*
Antidepressants (%)	31 (49)	12 (43)	.58
Benzodiazepines (%)	10 (16)	9 (32)	.1

*Note*. Eight patients without a proven PNES episode during Video‐EEG‐monitoring were not included. In Germany, patients with seizures, loss of consciousness, or similar conditions may not be allowed to conduct a vehicle for a certain time while still keeping their driver's license. If patients did not provide any information, included number is indicated for respective variables. Data are given as number and percentage (χ^2^ test or Fisher's Exact test) or median and interquartile range (IQR; Wilcoxon Rank Sum test). P‐values < 0.05 are labeled with asterisk (*).

Abbreviations: IQR,  interquartile range; PNES, psychogenic nonepileptic seizures.

### 3.4 Factors independently associated with PNES activity/cessation

The following relevant variables showing at least a trend toward an association with PNES cessation were included into the logistic regression model of both the complete and the sensitivity cohort: age at onset, time between symptom onset and diagnosis, tongue biting, dissociative status, motor symptoms. A reported history of a depressive/bipolar disorder was included into the sensitivity cohort model. A reported anxiety diagnosis and additional epilepsy were included into the model of the complete cohort.

#### 3.4.1 Total cohort (*n* = 99)

Older age at symptom onset (OR related to PNES cessation: 0.95 (95% CI 0.90–0.99)), additional epilepsy (OR 0.16 (95% CI 0.03–0.83)), reported anxiety disorder (OR 0.15 (95% CI 0.04–0.61)), and tongue biting (OR 0.22 (95% CI 0.04–0.91)) remained independently associated with ongoing PNES activity in the complete cohort. Time between symptom onset and diagnosis was not associated with PNES cessation after adjustment (OR 0.92 (95% CI 0.84–1.02)).

#### 3.4.2 Sensitivity cohort (*n* = 63)

Older age at symptom onset (OR related to PNES cessation: 0.89 (95% CI 0.82–0.97)), tongue biting (OR 0.002 (95% CI 0.0–0.1)), reported depressive disorder (OR 0.03 (95% CI 0.003–0.34)), and dissociative status (OR 0.06 (95% CI 0.007–0.51)) were independently associated with ongoing PNES activity (90% of patients classified correctly; Hosmer‐Lemeshow‐Test indicated a good model fit (χ^2^ = 6.24, *p* = .62); two patients were classified as outliers and excluded for the final sensitivity analysis (Hosmer et al., 2013; Lemeshow & Hosmer, 2008)). ROC analysis revealed 26.5 years as optimal cut‐off regarding the prediction of PNES cessation (Youden‐Index 0.41, sensitivity 0.71, specificity 0.69).

### 3.5 Interactions and subgroup analysis (sensitivity‐cohort only; *n* = 63)

Time between onset and diagnosis was prolonged in patients with a reported diagnosis of anxiety disorder (*n* = 25: median 7.3 years (IQR 2.7–13.1) vs. patients without anxiety disorder (*n* = 38): 3.0 years (IQR 0.9–9.3), *p* = .049) and patients with a tongue bite (*n* = 19; median 7.9 years (IQR 3.2–13.3) vs. *n* = 44; 3.2 years (IQR 0.9–9.6), *p* = .03) while this effect could not be shown in association with the diagnosis of a depressive disorder. A reported anxiety disorder was associated with increased odds for reporting the occurrence of a dissociative status (OR 4.6 (95% CI 1.6–13.6)). Patients with a depressive disorder (*n* = 46) reported more often a comorbid anxiety disorder (22 (48%) and 3 (18%), *p* = .04) and were older at symptom onset than patients without a depressive disorder (median 32 years (IQR 19–41) and 18 years (IQR 14–34), *p* = .01). Thus, we also analyzed the association of a depressive disorder with PNES cessation dependent on age: dividing the sensitivity cohort according to the cut‐off (26.5 years) estimated in the ROC analysis (see earlier), 35 patients were older than 26 years and 28 patients younger. Adjusted logistic regression revealed an association of a depressive disorder with ongoing PNES activity only in patients older than 26 years (OR related to PNES cessation 0.04 (95% CI 0.002–0.78)) while this effect could not be seen in patients younger than 26 years (OR 0.21 (95% CI 0.02–1.84)).

### 3.6 Importance of PNES cessation

In the total cohort, 57 (58%) patients considered PNES as their major medical issue. All but one patient (99%) considered PNES associated with QoL. QoL differed significantly between outcome groups. Patients with ongoing PNES activity were more often dependent on daily support, received more often governmental financial support, were less often employed, and showed a higher median percentage of severe disability. Those results were shown in both the complete and the sensitivity cohort (Table 1 and  3). Median QoL was 7, thus a “favorable QoL” was defined as = >7. After adjusting for reported depressive disorder and anxiety, PNES cessation independently predicted favorable QoL (sensitivity cohort: OR = 8.9 (95% CI 2.1–37.4); complete cohort: OR = 7.7 (95% CI 2.3–25.5)), while comorbid epilepsy was not associated with QoL (unadjusted OR = 1.03 (95% CI 0.43–2.47)).

**TABLE 3:** Outcome characteristics and patients’ current situation at the time of the interview

		Total cohort (N = 99)			Sensitivity cohort – PNES only (N = 63)		
	All patients (*n* = 99)	Ongoing PNES activity (*n* = 74)	No PNES activity ≥1 year (“PNES cessation”; *n* = 25)	*p*‐value (not corrected)	Ongoing PNES activity (*n* = 42)	No PNES activity ≥1 year (“PNES cessation”; *n* = 21)	*p*‐value (not corrected)
Quality of life (IQR)	6.5 (4.5–8)	5.5 (4–7)	8 (7–9)	<.001*	5 (3–7)	8 (7–9)	<.001*
Life without financial and ADL support possible (independent) (%)	56 (57)	35 (47)	21 (84)	.002*	19 (45)	17 (81)	.008*
Daily support (ADL) necessary (%)	37 (37)	37 (50)	0 (0)	<.001*	21 (50)	0 (0)	<.001*
No governmental financial support necessary (%)	35 (35)	21 (28)	14 (56)	.01*	11 (26)	13 (62)	.01*
Percentage of severe disability [%] (IQR)	60 (40–80; *n* = 92)	60 (50–90; *n* = 92)	40 (0–70; *n* = 92)	.004*	60 (50–90; *n* = 58)	40 (0–70; *n* = 58)	.008*
Number of anti‐seizure drugs (IQR)	0 (0–2; *n* = 98)	1 (0–2; *n* = 98)	0 (0–1; *n* = 98)	.01*	0 (0–2)	0 (0–1)	.39
Anti‐seizure medication (%)	47 (47; *n* = 98)	40 (55; *n* = 98)	7 (28; *n* = 98)	.04*	14 (33)	5 (24)	.56
Antidepressants (%)	44 (44; *n* = 98)	38 (52: *n* = 98)	6 (24; *n* = 98)	.02*	26 (62)	5 (24)	.007*
Benzodiazepines (%)	19 (19; *n* = 98)	19 (26; *n* = 98)	0 (0; *n* = 98)	.006*	10 (24)	0 (0)	.02*
Neuroleptics (%)	20 (20; *n* = 98)	18 (25; *n* = 98)	2 (8; *n* = 98)	.09	14 (33)	1 (5)	.01*
Number of other drugs (IQR)	1 (0‐3)	2 (0‐4)	1 (0‐1)	.04*	2 (0‐4)	0 (0‐1)	.007*
Number of patients with completed psychotherapy (behavioral or psychodynamic) (%)	42 (42; *n* = 98)	33 (45; *n* = 98)	9 (36; *n* = 98)	.49	22 (54; *n* = 62)	8 (38; *n* = 62)	.29
Number of patients with completed psychotherapies (including not further specified forms) (%)	55 (56; *n* = 96)	43 (60; *n* = 96)	12 (50; *n* = 96)	.48	30 (75; *n* = 60)	9 (45; *n* = 60)	.04*
Ongoing psychotherapy (%)	29 (29; *n* = 98)	26 (36; *n* = 98)	3 (12; *n* = 98)	.04*	16 (39; *n* = 62)	2 (10; *n* = 62)	.02*
Psychotherapy was considered effective (%)	27 (27; *n* = 97)	20 (27; *n* = 97)	7 (29; *n* = 97)	.99	14 (34; *n* = 61)	6 (30; *n* = 61)	.99
Psychotherapy was considered effective (first pass) (%)	8 (8; *n* = 47)	7 (19; *n* = 47)	1 (9; *n* = 47)	.66	5 (23; *n* = 31)	1 (11; *n* = 31)	.64
Drug therapy was considered effective (%)	6 (6; *n* = 97)	6 (8; *n* = 97)	0 (0; *n* = 97)	.33	3 (7; *n* = 61)	0 (0; *n* = 61)	.54
Combination of drug therapy and psychotherapy was considered effective (%)	12 (12; *n* = 97)	9 (12; *n* = 97)	3 (13; *n* = 97)	.99	6 (15; *n* = 61)	3 (15; *n* = 61)	.99
Regular neurological consults (%)	68 (69; *n* = 98)	56 (77; *n* = 98)	12 (48; *n* = 98)	.007*	32 (78; *n* = 62)	10 (48; *n* = 62)	.02*
Regular consults at an epilepsy center (%)	13 (13; *n* = 89)	12 (17; *n* = 89)	1 (5; *n* = 89)	.28	3 (8; *n* = 55)	1 (6; *n* = 55)	.99
Regular psychiatric consults (%)	38 (38; *n* = 98)	35 (48; *n* = 98)	3 (12; *n* = 98)	.002*	23 (56; *n* = 62)	2 (10; *n* = 62)	<.001*

*Note*. Total cohort (*n* = 99, left column); comparison of patients with ongoing psychogenic nonepileptic seizures (PNES) activity and PNES cessation in total cohort (*n* = 99; columns 2–4) and sensitivity cohort (*n* = 63; columns 5–7)). Sensitivity cohort includes only patients with proven PNES during Video‐EEG‐monitoring without additional epileptic seizures. Percentage of severe disability represents a measure of the German social insurance system to quantify patients’ need of and/or entitlement to payments or benefits. If patients did not provide any information, included number is indicated for respective variables. Data are given as number and percentage (χ^2^ test or Fisher's Exact Test) or median and interquartile range (IQR; Wilcoxon Rank Sum test). P‐values < 0.05 are labeled with asterisk (*).

Abbreviations: ADL, activity of daily living; PNES,  psychogenic nonepileptic seizures.

### 3.7 Current medical situation

In both cohorts, patients with ongoing PNES activity reported regular neurological and psychiatric consults more often than patients with PNES cessation (see Tables 1 and  3). About 75% of patients with ongoing PNES activity reported regular neurological contacts and about 50% reported regular psychiatric contacts. Less than 20% of patients (irrespective of PNES activity) reported regular consults at an epilepsy center. In patients without comorbid epilepsy, 33% of patients with ongoing PNES activity and 25% of patients with PNES cessation were still prescribed ASM. Patients with ongoing PNES activity reported a regular intake of antidepressants, benzodiazepines, and neuroleptics more often than patients with PNES cessation. Also, patients with ongoing PNES activity reported more often to having completed any form of psychotherapy and taking part in an ongoing psychotherapy. However, only few patients (irrespective of PNES frequency) considered any treatment effective so far.

## 4 DISCUSSION

Outcome in terms of PNES cessation over a period of 1 year prior to follow‐up was poor and seemed to matter regarding QoL and financial independency. Comorbid epilepsy, older age, comorbid psychiatric diseases (especially depressive disorders and anxiety), and semiology (tongue bite) were associated with ongoing PNES activity, which may reflect heterogeneous pathophysiological pathways: The association of comorbid psychiatric diseases with PNES activity seems to depend on patients’ age at PNES onset. Especially in patients with late‐onset PNES, comorbid depressive disorders seem to play an important role. On the other hand, patients with comorbid epilepsy reported comorbid psychiatric diseases less often while nearly all patients with comorbid epilepsy reported ongoing PNES activity. Diagnostic delay did not independently predict ongoing PNES activity in our cohort. However, especially symptoms suggesting an alternative epileptic or psychiatric diagnosis were associated with a delay in diagnosis. While in most patients with PNES cessation, PNES activity seems to stop within 1–2 years after diagnosis, efficacy of therapeutic options remains to be elucidated.

### 4.1 Outcome, PNES cessation, and predictive factors

Other studies also reported poor outcome in PNES patients (Reuber et al., 2003; Selwa et al., 2000). While some studies did not define “PNES cessation” or used a shorter period (3 or 6 months), we defined “PNES cessation” based on a period of at least 12 months without any PNES episodes. So far, no established predictive factors exist, as studies assessed different variables with sometimes conflicting results. Especially, comorbid psychiatric diseases (Lempert & Schmidt, 1990; Reuber et al., 2003; Walczak et al., 1995) and ongoing psychopathology (high levels of anxiety, depression, borderline personality, or dissociation) (Kuyk et al., 2008; Labudda et al., 2020; LaFrance et al., 2014) seem to be associated with PNES activity. We could confirm those associations while our results suggest an interaction with patients’ age at symptom onset. The ROC analysis revealed 26 years as optimal cut‐off regarding prediction of PNES cessation. In patients older than 26 years, a history of depressive disorder was associated with ongoing PNES activity, while this association was not present in younger patients. Other studies also concluded that underlying psychopathology may differ dependent on patients’ age at symptom onset with family distress, violence/abuse (Asadi‐Pooya et al., 2019), and a wide range of psychiatric disorders including adjustment disorders and neurodevelopmental disorders (Hansen et al., 2021) as predictive factors in children (Irwin et al., 2000). In adults, studies reported a correlation of improved psychopathology with reduced PNES frequency or cessation (Kuyk et al., 2008; LaFrance et al., 2014). However, some studies also found ongoing psychopathology in patients with PNES cessation, which was still associated with poor QoL and functioning (Lempert & Schmidt, 1990; Reuber et al., 2005; Walczak et al., 1995) questioning the reliability of PNES cessation as outcome variable. Our data suggest an association of PNES cessation with higher QoL and functioning even adjusted for psychiatric comorbidities. Other studies also found an association of PNES cessation or frequency with economic activity (Ettinger et al., 1999; Mayor et al., 2010), financial independency (Mayor et al., 2010), or QoL (Jones et al., 2010; Kuyk et al., 2008; Quigg et al., 2002). Finally, there seems to be a complex association of heterogeneous and age‐dependent psychopathological activities with PNES activity, QoL, and functioning with depressive disorders becoming more important in patients with late‐onset PNES.

Dramatic clinical features (loss of consciousness, urine/feces, tongue bite) have been described to be associated with unfavorable outcome (Reuber et al., 2003; Selwa et al., 2000). We found an association of tongue bite with ongoing PNES activity. Because we also found an association of tongue bite with loss of urine and feces, one may argue that those symptoms might be associated with undiagnosed epilepsy, even if this association has also been questioned before (Brigo et al., 2013; Brigo et al., 2012). We could not find an association of tongue bite with diagnosed epilepsy. Even if this may not exclude undiagnosed epilepsy, we could confirm the association of tongue bites with PNES activity in our sensitivity analysis including only patients with no hints of coexisting epilepsy. Thus, impressive clinical features may also represent severity and somatization of special patterns of psychopathology (Reuber et al., 2003; Reuber et al., 2003; Selwa et al., 2000) (e.g., anxiety), which might explain the association with PNES activity.

### 4.2 Diagnostic delay

There are conflicting results regarding the association of a shorter diagnostic delay with PNES cessation (Lempert & Schmidt, 1990; Reuber et al., 2003; Selwa et al., 2000; Walczak et al., 1995). Lempert et al. described a cut‐off of < 5 years as predictive regarding PNES cessation (Lempert & Schmidt, 1990). In our cohort, patients with ongoing PNES activities showed a median diagnostic delay of > 5 years  versus < 2 years in patients with PNES cessation. However, this difference did not independently predict PNES cessation. It is hypothesized that a short delay might be associated with a more active attitude of a patient toward medical examination and treatment (Bodde et al., 2012; Reuber et al., 2003; Selwa et al., 2000; Walczak et al., 1995), which may mediate the association with PNES cessation. On the other hand, Reuber et al. found an association of pathological EEG phenomena with diagnostic delay suggesting that treating physicians may also play a role (Reuber et al., 2002). We found an association of anxiety disorder and tongue bite with diagnostic delay suggesting difficulties in diagnosing PNES in patients with a relevant comorbid diagnosis or symptoms suggesting an alternative diagnosis.

### 4.3 Course of disease after diagnosis

Patients with PNES cessation frequently reported their last PNES episode within 1 (50%) to 2 (80%) years after diagnosis, even without proper treatment. Some authors considered the mere communication of the diagnosis an intervention (McKenzie et al., 2010) or concluded that patients’ appropriate understanding of PNES might be essential for a reduction of symptoms (Aboukasm et al., 1998). The way of reporting was not associated with outcome in our study. However, diagnosis was clearly explained in all cases. Thus, one of the described key elements associated with outcome may have been fulfilled in all our patients (Reuber & House, 2002).

### 4.4 Patients’ current situation

One‐third of patients without comorbid epilepsy still were prescribed ASM after the diagnosis of PNES. The proportion of those patients was even higher in patients with ongoing PNES activity. Other studies also reported comparable results (Reuber et al., 2003; Selwa et al., 2000). This may be due to a poor connection of patients with outpatient specialists (neurologists, epileptologists, and psychiatrists), as reported in our study and other studies (Selwa et al., 2000). However, safety of ASM withdrawal has poorly been studied (Duncan, 2006). In addition, the optimal management still needs to be elucidated. PNES patients may benefit from a comprehensive, multidisciplinary treatment following cognitive‐behavioral principles (Goldstein et al., 2010; Kuyk et al., 2008; LaFrance et al., 2014; Tolchin et al., 2019). A recent trial including 368 patients found no association of a cognitive‐behavioral therapy specific to PNES with PNES frequency at 12 months as primary endpoint (Goldstein et al., 2020). The authors reported a treatment association with the longest period without any PNES episode and psychosocial functioning, which might impact patients’ daily life. However, the included cohort was quite heterogeneous regarding age at onset and depression and anxiety‐related test scores at baseline (PHQ‐9 (depression), GAD‐7 (anxiety)). Furthermore, the authors reported problems with loss‐to‐follow‐up and uncertainties regarding the diagnosis of PNES and comorbid epilepsy: some patients were diagnosed without VEM confirmation and active epilepsy was ruled out on the basis of self‐reports. Another prospective study also found minor effects of psychotherapy at most while pre‐treatment psychopathology seemed to be the key prognostic factor (Labudda et al., 2020). Treatment in our study was not standardized and neither drug therapy nor psychotherapy was considered effective. In line with this, the percentage of patients with completed psychotherapy tended to be lower in patients with PNES cessation. However, treatment adherence was low in our cohort.

There are several strengths and limitations to our study. We retrospectively included a heterogeneous cohort and assessed multiple variables with multiple interactions. Furthermore, only patients admitted to an epilepsy center were included and half of the patients could not be included, which may pose a possible selection bias. Thus, an external validation may be needed. However, our cohort represents a rather large cohort compared to existing studies with well‐defined and controlled inclusion criteria and long‐term follow‐up. Furthermore, we performed a sensitivity analysis in patients with confirmed “PNES only.” Data were obtained by questionnaire or telephone interview. However, we verified as much data as possible using medical records or contacting family physicians. The diagnosis of comorbid epilepsy was verified on the basis of our EEG recordings. We could not obtain sufficient information regarding the location of the tongue bite (tip of tongue vs. lateral) to further assess the association with epileptic seizures and PNES. We included reported psychiatric diagnoses, which patients had received at any time. Results regarding ongoing psychopathology and personality profile are reported elsewhere (Walther et al., 2019).

## 5 CONCLUSION

Patients should be followed up closely using an interdisciplinary approach also addressing ongoing psychopathology and treatment adherence, which may improve treatment efficacy. Special attention should be paid to patients with comorbid epilepsy. Prospective studies with regular diagnostic epileptic and psychiatric workup to reassure diagnosis and assess interactions are warranted.

## SOURCES OF FUNDING

None.

## DISCLOSURES

B. Volbers reports personal fees from Pfizer AG/Bristol‐Myers Squibb SA, personal fees from Bayer AG, grants from Institutional grant (Inselspital), personal fees from Ipsen Pharma, personal fees from CSL Behring, outside the submitted work; K. Walther reports no disclosures relevant to the manuscript; K. Kurzbuch reports no disclosures relevant to the manuscript; L. Erdmann reports no disclosures relevant to the manuscript; S. Gollwitzer reports no disclosures relevant to the manuscript; J.D. Lang reports no disclosures relevant to the manuscript; M. Dogan Onugoren reports no disclosures relevant to the manuscript; M. Schwarz reports no disclosures relevant to the manuscript; S. Schwab reports no disclosures relevant to the manuscript; H. M. Hamer has served on the scientific advisory board of Angelini, Corlieve, Eisai, GW, Sandoz, UCB Pharma, and Zogenix. He served on the speakers’ bureau of or received unrestricted grants from Ad‐Tech, Bracco, Desitin, Eisai, GW, Micromed, Nihon Kohden, Novartis, Pfizer, and UCB Pharma.

## TRIAL REGISTRATION

There was no trial registration because this study does not represent a clinical trial according to the ICMJE‐definition.

### PEER REVIEW

The peer review history for this article is available at https://publons.com/publon/10.1002/brb3.2567.

## Data Availability

The data that support the findings of this study are available from the corresponding author upon reasonable request.
